# Delineating connectivity and quality of peer–peer pre-pubescent rhesus macaque (*Macaca mulatta*) relationships, by examining coupled social behaviours

**DOI:** 10.1098/rsos.250860

**Published:** 2025-10-22

**Authors:** Alexander J. Pritchard, Tyler Bonnell, Bidisha Chakraborty, Rosemary A. Blersch, Brenda McCowan, Jessica J. Vandeleest

**Affiliations:** ^1^ Department of Population Health and Reproduction, School of Veterinary Medicine, University of California Davis, Davis, CA, USA; ^2^ Neuroscience and Behavior Unit, California National Primate Research Center, Davis, CA, USA; ^3^ University of Calgary, Calgary, Alberta, Canada

**Keywords:** social development, multilayer networks, laboratory animals, primates

## Abstract

Infant experiences have lifetime implications for individuals’ social competence. Therefore, lifelong trajectories can be informed by a nuanced understanding of *who* developing individuals connect with (i.e. connectivity) and *how invested* they are in those connections (i.e. quality). Though simple in premise, the practice of examining social connectivity and quality relies on a nuanced understanding of how individuals temporally shift their behavioural repertoire within, or across, partners. We measured peer–peer relationships throughout the first 3 years of life among 49 rhesus macaques (*Macaca mulatta*) in large outdoor-housed mixed-sex home groups. We recorded five social behaviours and built multiplex temporal networks. We examined the auto- and cross-correlations of these behaviours using a multivariate multiple response time series model to understand the behavioural dynamics of relationship connectivity and quality. We demonstrate known principles of relationship formation driven by pre-pubescents’ and peer partners’ traits (i.e. rank, sex, age, kinship). Coupled dynamics suggest that proximity was broadly associated with social dynamics (including aggression), while contact was associated with prosocial dynamics (excluding aggression). Directed behaviours were less associated with each other. These results highlight the dynamic nature of social development across multiple behaviours, underscoring how early social choices shape the formation, stability and maintenance of relationships.

## 1. Introduction

Social relationships are complemented by a diverse array of complex behavioural exchanges and social cognitive processes. Among humans and other primates, relatively protracted development time has been posited to afford such social, cognitive and behavioural complexity [1]. Yet, the developmental process necessitates navigating a socially complex system at an early age. What are the ‘rules of engagement’ during social development, and how do they shift across behaviours? How do individuals prioritize both *who* they connect with (i.e. connectivity) and *how invested* they are in those connections (i.e. quality)? These prioritizations need not be congruent in their motivating factors [2]. Resolving these questions is of importance given the lifetime implications of social learning, and the disproportionate importance of pre-pubescent development and adversity [3].

In rhesus macaques (*Macaca mulatta*), as a translational model for humans, infant relationships change even in the first 30 weeks of life [4]. Pre-pubescent social development is often understood through the lens of measuring and interpreting play behaviour. Play has been posited as a functional primer for social competence [5]. Research on play has shown that: individuals differ in their quality of investment across partners, animals exhibit selectiveness in their connectivity and there are age-related changes in play [6–8].

Play is not the single defining behaviour of pre-pubescent relationships nor the first to emerge. Social proximity and contact are both early emergent social behaviours [7,9]. Proximity increases the probability of diverse social interactions [10], while contact is a key provisional behaviour for comfort and support [11]. Beyond proximate benefits, infants are more likely to form stronger quality bonds with individuals that engage in greater contact with them at an earlier age [9]. Even heightened early contact with human caregivers has been associated with improved motor, social and cognitive skills [12].

No single behaviour or context is sufficient to capture the reality [10] or abstraction of social connectedness. This is, in part, because social dynamics are spatio-temporally coupled via auto- and cross-correlations. For instance, past grooming and play have been shown to predict future associations in spatial proximity [13]. Furthermore, social behaviours are not necessarily uniformly influenced by the characteristics or traits of an individual. For example, despite early sex differences in prosocial behaviours [7], aggression did not show sex differences [14]. These complexities necessitate analytical approaches that acknowledge and synthesize these complications as intrinsic properties of the system.

Infants are expected to exhibit differences in sociality associated with their intrinsic or maternal traits. For instance, sons have been observed to receive greater maternal grooming than daughters [15]. Infants with higher ranking mothers have been shown to be more connected in their peer contact networks [16]. Younger wild rhesus macaques generally exhibit a greater diversity of play and approach partners (i.e. higher connectivity) than subadults and adults, respectively [17]. Likewise, as individuals near pubescence the frequencies of grooming and aggression increase, while play decreases [7,13,14].

The influence of infant traits, however, is mediated through their partner’s traits. Such a dynamic can be typified via social assortativity, whereby individuals prefer to associate with conspecifics that exhibit similar (homophily) or dissimilar (heterophily) traits—as opposed to disassortativity where individuals do not exhibit such preferences for similarity or dissimilarity [2,18,19]. Among pre-pubescent rhesus macaques, female affiliative interaction partners are preferred, with sex homophily among infant females and heterophily among male infants [7]. After 2 years of age, however, males exhibit sex homophily [7]. Individuals exhibit increased preference to associate and play with kin, relative to non-kin [4,7,8,20]. Both males and females exhibited sex and age homophily for aggressive behaviours [14], as well as sex and rank homophily for play [8]. Older juveniles may, however, prefer to play with younger infants—though play behaviour decreases with age [8,21].

We sought to explore the dynamics of social relationships in two cohorts of pre-pubescent rhesus macaques from birth up to 3 years of age. We focused on peer networks, as age peers are preferred social partners [7,22]. Indeed, infant rhesus macaques show a tendency to seek peers [23]. Even so, the role of peers on socio-behavioural development is understudied [1]. We focused on connectivity and quality of social connectedness. Connectivity was a measure of whether a focal subject interacted with a given conspecific. Quality was a measure of a focal subject’s relative investment in a given conspecific. Despite numerous publications on social development, few simultaneously and explicitly consider relationship connectivity and quality as a dynamic process coupled across multiple behaviours—especially with aggression [22].

This conceptual approach provides an important step towards synthesizing relationship development in infants. Social relationships function as temporally dynamic processes that can, at least in principle, shift in the types of behaviour that are used for relationship maintenance over time. This expectation is especially true during periods of significant developmental transition (e.g. infancy or pubescence), when individual expression and social engagement might be expected to change. Additionally, there are co-dependencies between behaviours such that social partners can be represented across multiple behaviours and temporal periods. If developing animals shift their social investment across behaviours, then, from a univariate perspective, this might be perceived as downregulating or culling a relationship. Put simply, relationships are multivariate processes, a point which is not new [24–27] yet has not been fully integrated into conceptual and methodological practice. Thus, we aimed to quantify how social relationships, measured via connectivity and quality, might remain stable across and within each of five social behaviours: aggression, contact sitting, directed play, grooming and proximity. This approach incorporates the temporal dynamics of relationships, multivariate behaviour as a property of social relationships, and whether a bond exists (connectivity) as well as the relative investment in that bond (quality). This conceptual approach is facilitated through a reliance on contemporaneous methods that facilitate temporal network structures and multivariate response models to enable insight into social relationships as a multivariate and dynamic coupled process.

### 1.1. Hypotheses and predictions

This work straddles a problem-focused approach, substantiated by a number of publications on infant social development, and an exploratory approach, whereby little is known about how relationship connectivity transitions across numerous behaviours in peer networks. Our methodological and analytical approach facilitates a new way to approach understanding infant development, as maturing individuals are expected to shift social investment across conspecifics and among behaviours as they change in prevalence across development. In summary, these analyses are predominantly exploratory, with our predictions selected to highlight relevant prior works and provide scaffolding for our findings. In practice, much focus has been dedicated to assortativity and the associations between social behaviour and maturing individuals’ or their mothers’ traits. The interconnected dynamics of social behaviours and the distinction between connectivity and quality during development are poorly described, in comparison.

#### 1.1.1. Coupled behavioural dynamics

We had general expectations regarding the associations between the five behaviours. Assuming proximity increases the likelihood of all other behaviours [10], then proximity in the present and preceding time periods should be associated with connectivity and quality of all behaviours. Social contact was expected to meaningfully precede the development of play [9,12], while play would meaningfully precede grooming and aggression [5].

#### 1.1.2. Ego traits

We hypothesized that the infant subjects’ (i.e. egos’) traits would be relevant to their social connectivity. We expected that higher ranked subjects would have higher social connectivity across social behaviours except aggression, relative to lower ranked subjects [7,16,28]. Establishing clear predictions for aggression was more challenging, because we simplified to undirected aggression. This simplification was because our interest was in general social relationships—not autonomy or agency of maintaining those relationships; additionally, to avoid doubling the number of response variables for directed behaviours. We anticipated, however, that low-ranked individuals might be more likely to avoid aggression or mothers might be more likely to extract infants if circumstances are agonistically escalating. Thus, high ranking infants would be expected to have greater connectivity.

Sex differentiation can emerge within six months [6,7,22]. Male subjects would be expected to have higher connectivity in play networks [7,28], while female subjects will have greater connectivity in grooming [7]. Though grooming and aggression behaviours were expected to increase with age, while play was expected to decline [7,14]. For contact and proximity, we expected partner selectivity to be more pronounced as individuals age [17]. Thus, we anticipated that contact and proximity connectivity would decline with age. Though mothers are more protective of younger infants [11], this effect would be expected to decrease as individuals age into the pre-pubescent stages that defined the majority of our study.

#### 1.1.3. Assortativity

We expected that subjects would exhibit sex, rank, kin and age homophily in aggression [4,7,8,14,21,29]; rank, kin and age homophily in grooming, proximity, play and contact [4,7,8]; and sex homophily in play [6–8,11]. Although older pre-pubescent females interact with new infants [8,21], it is unlikely this would be majority represented in our dataset.

## 2. Methods

### 2.1. Subjects

Our study included 49 infant subjects (27 females; 22 males) observed in their home group over their first 3 years of life. These subjects came from two birth cohorts (2016 and 2017) with the study’s duration extending from 2016 to 2019. The home group consisted of a single outdoor-housed mixed-sex group of rhesus macaques at the California National Primate Research Center (CNPRC). The group had a mean size of 133.5 animals (range = 120–156) with a mean F:M sex ratio of 2.05 [30–32]. Animals were housed in a half-acre enclosure with A-frame structure, perches, benches, free access to food and water, heaters, sprinklers, play structures and shade structures. Housing, animal husbandry and data collection protocols were approved by the Institutional Animal Care and Use Committee at the University of California, Davis.

### 2.2. Observations

We completed focal observations on subjects in their group’s outdoor enclosure for 10 min, twice a week, balanced across mornings and afternoons. We recorded subject social behaviours using 30 s intervals. State behaviours (contact-sit [contact, hereafter] and proximity) were sampled on the 30 s mark, while the remaining directed behaviours (directed play [play, hereafter], grooming, and aggression) were scored as a 1/0 for unique dyads interacting during each 30 s interval—with the dyadic direction recorded. Play was only coded for active interactions with a social partner that included non-aggressive chasing, bouncing, tumbling, grabbing, wrestling, soliciting or mock biting, often with an open mouth play face. Observers surpassed an inter-observer reliability threshold of Krippendorf’s alpha ≥ 0.85 for behavioural coding.

We also conducted event sampling to record instances of status signalling and dyadic aggression among all group members. These data were collected 3 days a week throughout the project. We used these data to assemble linear social dominance hierarchies within each of the four study years.

### 2.3. Extracting social measures

#### 2.3.1. Ego temporal network centralities

**Figure 1.**
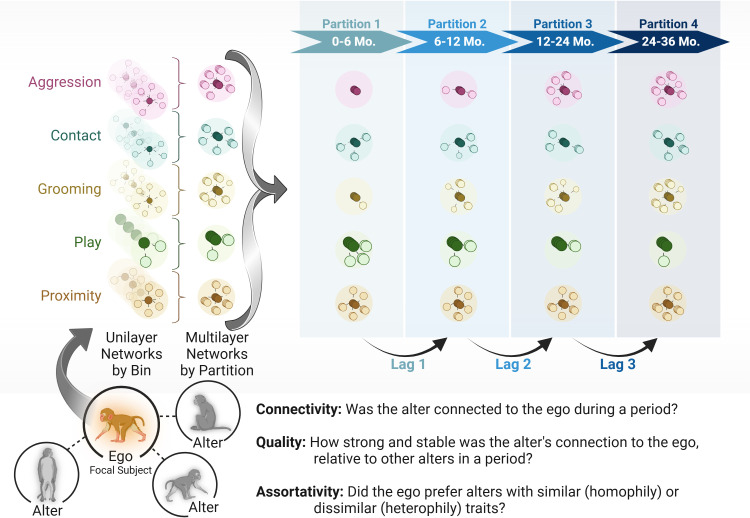
Conceptual diagram of the data processing (also see electronic supplementary material, text S1), analytical approach and central questions for our project. Ego networks centred on our infant subjects were constructed in monthly bins, for five behaviours. These unilayer networks were partitioned into four multilayer networks, each representative of a different developmental time period. These multilayer networks were then used to calculate connectivity and quality of egos’ connections (alters), to assess the influence of egos’ traits, alters’ traits relative to the ego (assortativity), as well as how connectivity and quality of these five behaviours were interconnected during development. Created in BioRender (https://BioRender.com/p04e956).

Briefly, we assembled temporal multilayers for each of the focal subjects (egos, hereafter) across the five behaviours to extract multilayer versatility as a measure of connectivity and quality (figure 1). This process was initiated by collating each egos’ focals into unique monthly bins that contained a unilayer network for one of the five behaviours. We refer to these networks as unilayer because they represented a single behaviour within a single month of association data. They are ego networks as they are defined by the focal subject’s connections with no connectivity between the ego’s social partners (alters, hereafter), resulting in ego-centred star networks. We excluded alters that were more than 3 years old in the respective year of birth for each cohort. Thus, throughout the remaining processing, the alters were between 0 and 6 years of age. Data processing during these aforementioned steps is further detailed in electronic supplementary material, text S1. We aggregated these bins into four discrete time periods each containing 5−6 bins (months) of data. Focals were collected every month in the first year, and every other month thereafter. Subjects had 7.68 M ± 1.32 sd focals per bin. These aggregations generally represented the relative age of the subjects, such that partition one included 1−6 months, partition two included 7−12 months, partition three included data from year one, and partition four included year two. This process resulted in 980 potential temporal ego networks (49 [subjects] × 5 [behaviours] × 4 [partitions]). One subject was missing their final partition and not all behaviours were expressed in each partition (i.e. rates of grooming were generally low among infants). Thus, the number of realized temporal networks was 947 (225, 242, 241 and 239 in partitions one, two, three and four, respectively). The number of unique ego-alter dyads were 3871, 4830, 5126 and 4575 in partitions one, two, three and four, respectively. Same sex dyads made up 51.54%, 52.15%, 52.11% and 51.65% of the connections for partitions one, two, three and four, respectively.

We calculated PageRank versatility (versatility, hereafter) for each node in the temporal networks [33,34]. Versatility measures each node’s importance across layers (i.e. our temporal bins) and within each layer (i.e. relative centrality compared to other nodes in a given bin). This capacity is attributable to the use of a random walker that can move within layers, but also across layers. Because the multilayer networks are temporal aggregations, stability and strength (in a single partition or period of time) are both considered by PageRank versatility [33]. That is, a strong connection within a layer or connections to the ego among many layers could amplify an alter’s versatility—with the highest scores having both qualities (high intra-layer strength and high inter-layer ego connectivity). Therefore, we can obtain the alters in which those egos heavily invested in, even with regards to stable quality over time. We normalized versatility, which reduces the impact of our third partition’s decreased bin count (see electronic supplementary material, text S1) and differences in degree across ego subjects. Because there were no connections between alters, these indices are a measure of how connected each alter is to the ego. The ego’s versatility measures were excluded from their own multilayer ego networks. All animals were in the same enclosure and could have had contact with any one of their conspecifics during periods that they were co-present. Thus, we zero-populated PageRank scores for alters in any behaviours and partitions that they were not represented in, assuming they were present in other partitions and behaviours. This process was eminently logical to distinguish putative connectivity (zeroes) from realized connectivity (non-zeroes). We removed zeroes for alters before they were born, or after they were removed. This process resulted in multilayer PageRank values for each of the five behaviours with all feasible dyads within each of the four partitions.

#### 2.3.2. Social dominance ranks

We calculated adult’s social dominance ranks for each year of observation (electronic supplementary material, text S2) and assigned each ego focal subject their maternal social dominance rank. Alters were assigned their own rank. If alters were too young for their own rank, then they were assigned the maternal or maternal kins’ rank.

#### 2.3.3. Temporal lags for behavioural dynamics

We computed a predictor variable from each behaviour lagged by a single partition to determine whether behaviours were autocorrelated or intercorrelated throughout time. That is, the first partition was included in the dataset as a lag for the second partition, the second for the third, and the third for the fourth. Our model excludes data from the first partition in the response variable because it has no lag variables.

#### 2.3.4. Sociodemographic differences between ego and alter for assortativity

We calculated the absolute difference in our rank estimates and age, in years, for all the egos and their alters. We included a dichotomous factor variable for whether the ego and alter were of the same sex or not. Finally, we included a factor variable for whether alters were: unrelated, co-matrilineally related but not siblings, or siblings.

#### 2.3.5. Other demographic qualities

We included factors for newly born or removed individuals. We assigned a dichotomous factor to flag if subjects were born during the previous partition. Because we relied on temporal lags, this dichotomous flag was assigned to the partition after the first lag that an alter was present for. Similarly, we assigned a dichotomous factor to flag alters that were removed during any given partition. We included cohort of the ego individuals as a factor to account for idiosyncrasies in annual group condition or normative behaviour.

### 2.4. Model construction

Our final dataset included 12 967 rows of ego-alter dyads. Versatility measures were non-zero for 60.89% of proximity dyads, 17.06% of contact dyads, 17.31% of play dyads, 13.30% of aggression dyads and 4.15% of grooming dyads. During model construction, we noted very few samples between zero and one for the grooming, aggression and play versatility measures. Thus, to reduce the complexity of our multivariate response model and facilitate interpretability, we converted all grooming, aggression and play versatility measures that exceeded 0 to 1. Because we had to simplify our response variables for the directed behaviours, the binomial models focus only on social connectivity in the response variable, but the lag variables retain information on connectivity (non-zero values) and quality. For these directed behaviours, versatility is not directional, but was calculated using directed temporal networks such that, assuming equal edge weights, alters with bidirectional connectivity to an ego would have higher versatility than those with unidirectional ties.

To quantify the coupled dynamics between the five behaviours across time we ran a multivariate response Bayesian regression model using Stan (brms) [35,36] in R version 4.4.1 [37]. Contact and proximity were fit using the hurdle-lognormal family with the response variable of versatility, while grooming, aggression and play were fit using Bernoulli distributions each with their respective binary response variable. Thus, the hurdle components of our multivariate model and the Bernoulli models test connectivity: whether or not dyads socialized. The lognormal components of the contact and proximity models predict the quality of investment among dyads that had connectivity; i.e. PageRank versatility which is proportional to each ego’s investment within that partition. A multivariate response model facilitates quantification of the magnitude of covariation that random effects exhibit across each of the response variables. All five response variables had the same model structure, and all continuous predictors were centred and scaled by two standard deviations [38]. Our model predictors included: the ego’s birth cohort, sex and maternal rank; the ego-alter’s rank (or, rather, the ego’s maternal rank relative to the alter’s individual or maternal rank), age and sex difference, as well as their relatedness; whether the alter was born or removed recently; the partition as a linear predictor; and five lagged variables for each of the behaviours. We included ego and alter subjects as random effects.

We ran 4 MCMC chains, with 6000 iterations, a warmup of 2000 and a thin of 2, resulting in 8000 post-warmup draws. We used weakly informative priors (Normal, 0,1) for the fixed effect terms (‘b’). Model fit was good based on $\hat{R}$ estimates, effective sample size estimates, Pareto k estimates (electronic supplementary material, text S3), posterior predictive check density plots (electronic supplementary material, figures S1–S5) and pairs plots (electronic supplementary material, figures S6–S12).

## 3. Results

Prior to parsing our model, we provide a more holistic presentation of the age-related trajectories for our five behaviours by presenting exploratory models for several common measures. This provides comparability for measures that are commonly understood, or not fully represented in our model structure before we examine the major outcomes of our full model.

### 3.1. Temporal dynamics of behaviours

**Figure 2.**
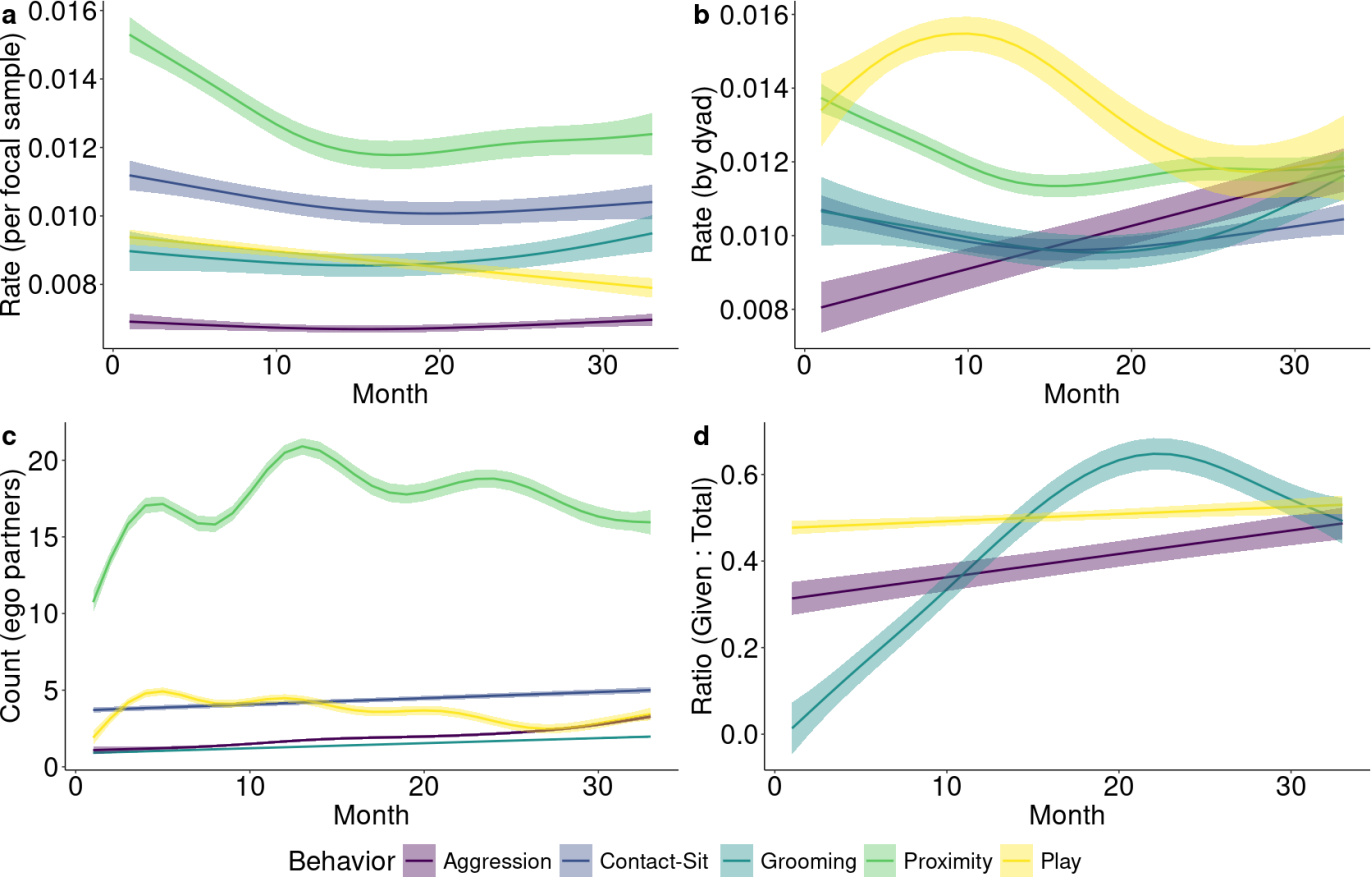
Rates of behaviour aggregated by month (a) within each focal and (b) within each dyad. We also present (c) the count of alter partners each ego had, aggregated by month. Finally, we present (d) the ratio of given : total directed behaviours calculated by the dyadic balance of given to total (i.e. given and received) for each behaviour, aggregated by month. Shading is indicative of the standard error.

We visualized the monthly ego networks to interpret overall developmental trends in the five social behaviours. To accomplish this, we built simple generalized additive mixed models (GAMMs) with ego focal as a random effect, and month as a fixed effect predicting: rates aggregated by ego focal (figure 2a), dyadic rates (figure 2b), partner counts (figure 2c) and ratio of behaviour given-to-total (figure 2d). This step was an important companion analysis to interpret our full model because our model: included gross age-related differences at the level of the dyad (connectivity) and normalized some variation prior to modelling directed behaviours (quality).

Aggression exhibited an uncertain (i.e. relatively high error) decline in focal rates in the first year of life and an uncertain increase after month 20 (figure 2a), but a continuous increase in rates aggregated by dyad (figure 2b). This latter increase corresponded to an increase in the average number of alters (figure 2c) and an increase in the average proportion of aggression given, relative to total aggression (figure 2d).

For contact, focal and dyadic aggregated rates showed an initial decline, bottoming out around month 15 (figure 2a,b). Dyadic rates of contact continued to increase after this initial decline, approximating a u-shaped dynamic. The number of contact alters showed a negligible increase over time (figure 2c).

Grooming rates, by focal, were consistent across the project period until an increase in, approximately, the last six months (figure 2a). When aggregated by dyad, we observed a slight uncertain decrease (i.e. with high error) followed by the same aforementioned increase (figure 2b). The number of grooming partners showed a slight increase across the entire project period (figure 2c). Individuals received more grooming than they gave for the first 10 months of life (figure 2d). The proportion of grooming given increased steadily through this time and until individuals typically gave more grooming than they received, around month 20. Towards the end of the project, this proportion decreased nearing reciprocity (0.50).

Play exhibited a decrease in focal rates (figure 2a). For dyadic rates (figure 2b), play exhibited an increase from birth to approximately month 10. Dyadic rates then declined until approximately month 24, at which point they stabilized. The count of alters followed a similar growth trajectory (figure 2c). Play approximated 0.5 in the ratio of given versus total play throughout the study, with a slight increase throughout the study period (figure 2d).

For proximity, the overall rates decreased over time before stabilizing around the mid-point of the study period (month 15). This pattern was true for the total rate of behaviours by the ego focal (figure 2a), and when rates were calculated by dyad (figure 2b). This decrease was despite an increase in the number of proximity partners, which peaked just prior to the mid-point of the study period (month 12) before decreasing (figure 2c). We note that newly born alters could be represented as early as month 12 and 24 and could be attributed to the ‘bumps’ at these times.

### 3.2. Modelled outcomes

#### 3.2.1. Variance explained

Our Bayesian *R*^2^ estimates (*bayes_R2* in brms) (table 1) indicated that our model varied in its capacity to explain our data’s variance across behaviours. Our two hurdle-lognormal response variables, contact and proximity, had the highest proportion of variance explained by the fixed effects: 52% and 64%, respectively. Grooming and play connectivity had 18% and 28% explained, respectively. Aggression connectivity had the lowest proportion of variance explained at 5%. Our fixed effects generally explained a high majority of model variance, as indicated by small differences between our conditional and marginal *R*^2^ estimates (|range| of Δ between *R*^2^_*C*_ and *R*^2^_*M*_ = 0.007 to 0.029).

**Table 1.** Bayesian *R*^2^ estimates and their uncertainty.

	conditional bayes *R*^2^ (*R*^2^_*C*_**)**			marginal bayes *R*^2^ (*R*^2^_*M*_**)**		
	estimate ± error	quantiles		estimate ± error	quantiles	
		2.5%	97.5%		2.5%	97.5%
aggression	0.07 ± 0.01	0.06	0.08	0.05 ± 0.01	0.04	0.06
contact	0.53 ± 0.01	0.50	0.56	0.52 ± 0.02	0.48	0.55
grooming	0.21 ± 0.01	0.19	0.24	0.18 ± 0.02	0.15	0.22
play	0.30 ± 0.01	0.29	0.32	0.28 ± 0.01	0.25	0.31
proximity	0.64 ± 0.01	0.63	0.66	0.64 ± 0.01	0.62	0.65

#### 3.2.2. Whole model results

After validation (electronic supplementary material, text S3, figures S1–S14), we turned our analytic focus to understand the models’ fixed effects as they pertained to each of our hypotheses and predictions. We emphasize that high estimates in the binomial distributions (aggression, grooming, play) indicate connectivity, while low estimates with the hurdles (contact, proximity) indicate connectivity—because positive movement in the hurdle indicates an increased probability of attaining a zero (no connectivity). For relevant figures, however, we reverse coded the hurdles to enable more intuitive comparability. For quality, higher values indicate higher proportional ego investment with that alter. Where possible, we communicate the direction of the socio-biological outcome (i.e. greater connectivity) rather than the directional influence of the estimate (i.e. reduced the probability of attaining a zero). See electronic supplementary material, table S1 for the full model summary.

##### 3.2.2.1. Coupled behavioural dynamics

**Figure 3.**
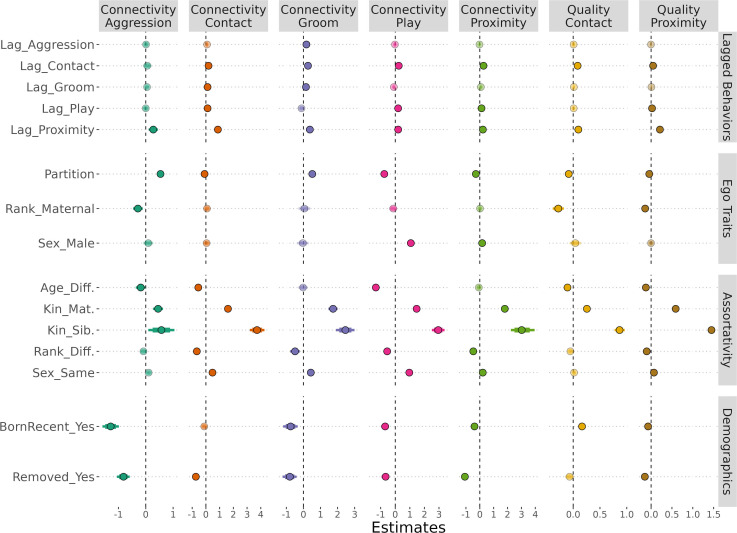
A summary of the fixed effect estimates across the five behaviours (column panels and colour). Hurdle response variables have been reversed for comparability (1.0 = connectivity, 0.0 = no connectivity). Estimates with 95% CIs that do not cross 0 (dotted vertical line) are emphasized with a darker hue. Row panels are added to organize effects by major themes from the manuscript.

All of our lagged variables had either positive or null associations to connectivity or quality, none had negative associations with connectivity or quality (figure 3). As expected, lagged proximity was predictive across all behaviours. Contact predicted play, as well as all other variables except aggression. Contrary to our prediction, play did not meaningfully predict grooming.

Here, we break the lagged associations down by response variables. For aggression, only lagged proximity predicted increased connectivity. For contact, increased connectivity was associated to lagged proximity and, to a lesser extent, lagged contact, grooming, as well as play. For grooming, increased connectivity was associated with lagged proximity, contact, aggression and grooming. For play, increased connectivity was associated with lagged contact, play and proximity. For proximity, increased connectivity was also associated with lagged contact, play and proximity. Contact quality was positively associated with lagged contact and proximity. Proximity quality was positively associated with lagged contact, play and proximity—though lagged play had high uncertainty approaching zero. To confirm that the lagged associations were not overly informed by a particular partition, we also executed models using a subset of data for each partition. The model formulas were identical, though Partition 2 had no subjects born the year prior, so this term was omitted. Generally, model estimates were directionally consistent, indicating that no partition drastically violated the broad patterns we observed (electronic supplementary material, figure S15).

**Figure 4.**
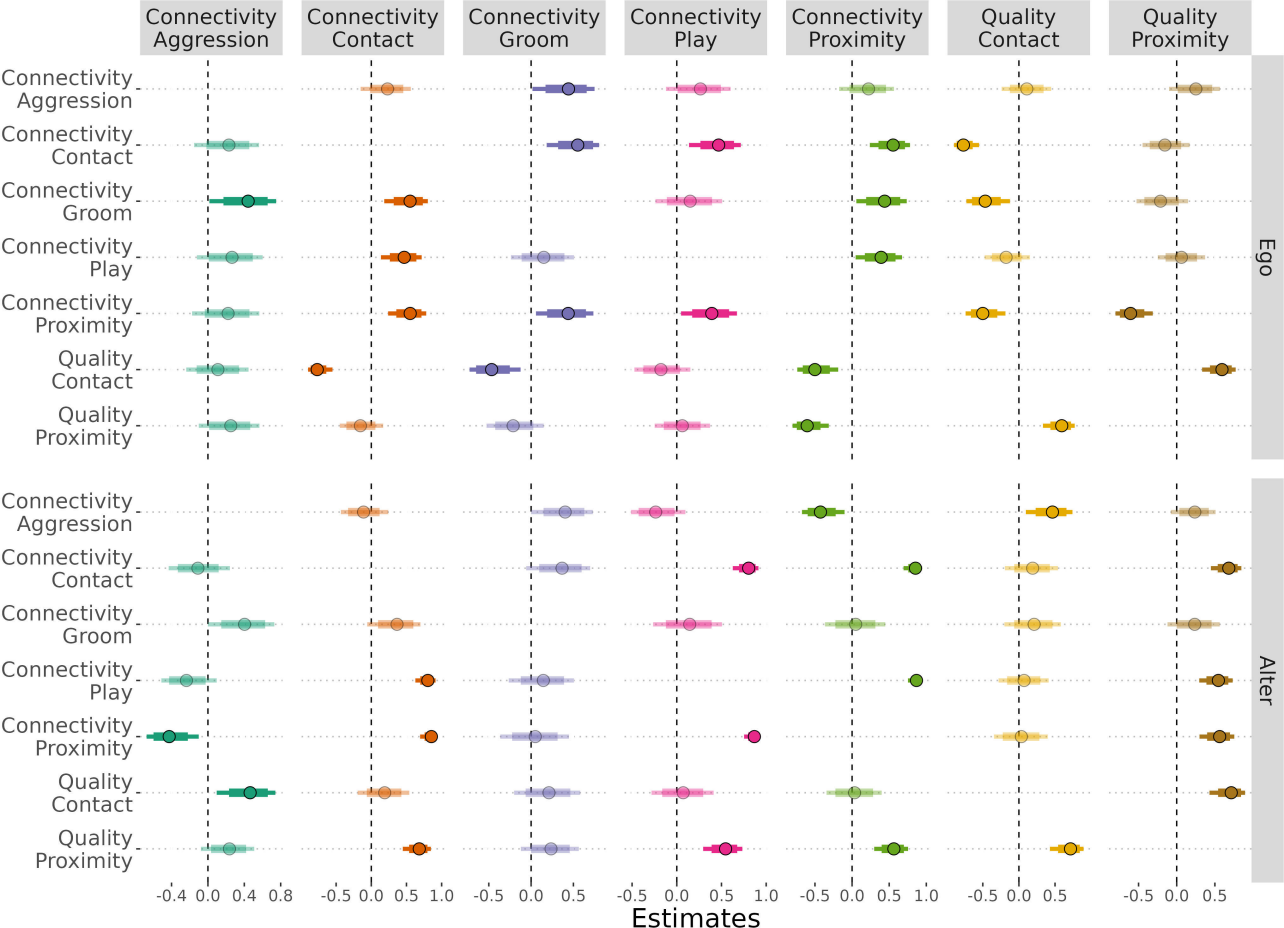
A summary of the cross-correlation results for egos’ (top panel) and alters’ (bottom panel) random effects across the five behaviours (column panels and *y*-axis). Hurdle response variables have been reversed for comparability (1.0 = connectivity, 0.0 = no connectivity). Colour is used to emphasize behaviour in the column panels. Estimates with 95% CIs that do not cross 0 (dotted vertical line) are emphasized with a darker hue. Cross-correlations are symmetrical such that the upper triangular is equivalent to the lower triangular, self-correlations are not reported.

To examine patterns of consistent individual differences of ego and alter subjects across behaviours, we examined the cross-correlations of random effects. Overall, the variation between egos’ (intrinsic) random effect estimates differed across the behaviours, though the certainty of these estimates did not markedly vary across individuals (electronic supplementary material, figure S16). Cross-correlation in the egos’ random effects suggested that individual differences in contact connectivity were positively correlated with individual differences in grooming, play, as well as proximity connectivity (figure 4). Similarly, we found individual differences in proximity connectivity that were positively correlated with individual differences in: grooming, play and contact, but negatively correlated to individual differences in contact quality. Interestingly, individual differences in aggression, grooming and play were not cross-correlated—with the exception of a weak positive correlation between grooming and aggression connectivity. Finally, individual differences in contact quality were negatively correlated to grooming connectivity, and positively correlated with proximity quality. To confirm that the fixed effects were not skewing the interpretation of our multivariate cross-correlations, we constructed two models with just our random effects, and just the lagged behaviours with random effects. Estimates for cross-correlations were similar between the full model and the two simpler models (electronic supplementary material, figure S17).

The variation between alters’ (extrinsic, relative to the ego) random effect estimates also differed across the behaviours, with play connectivity exhibiting the greatest interindividual variation. The certainty of the alters’ estimates were more highly varied for play and proximity connectivity (electronic supplementary material, figure S18). Cross-correlations in the alters’ random effects exhibited positive associations between contact connectivity and: play, and proximity connectivity as well as quality (figure 4). Proximity connectivity was similar to play connectivity and inversely related to aggression connectivity. The connectivity of aggression, grooming and play were not cross-correlated. Contact quality was positively correlated with aggression connectivity and proximity quality. Proximity quality was also correlated with play connectivity.

##### 3.2.2.2. Ego traits

We examined several traits linked to the ego: the partition as a proxy for age, maternal rank and sex (figure 3). Our findings generally followed our expectations for ageing; and, that males would have higher play connectivity. Maternal rank did not clearly adhere to our expectations. Using partition as a proxy for ego’s age, connectivity increased for aggression and grooming, but decreased for contact, play and proximity. Quality also decreased with age for both contact and proximity. Ego sex was associated with play and proximity such that males had higher connectivity relative to females. Finally, egos’ higher maternal rank was associated with lower aggression connectivity, and lower quality for both contact and proximity.

##### 3.2.2.3. Assortativity

We examined whether our pre-pubescent ego subjects exhibited assortativity in their connectivity and quality (figure 3). In line with expectations, homophily was a strongly governing principle throughout the majority of our behaviours. Egos and alters more similar in age (i.e. homophily) were more likely to have connectivity in aggression, contact and play, as well as higher contact and proximity quality. Any form of kinship (sibling or co-matrilineal) increased connectivity and quality across all five behaviours with no exceptions. Relative to co-matrilineal relationships, siblingship resulted in heightened connectivity or quality for all behaviours, with the exception of aggression and grooming connectivity. Egos and alters similar in rank (i.e. homophily) were more likely to have connectivity in contact, grooming, play and proximity, as well as higher proximity quality. Finally, egos and alters that were the same sex (i.e. homophily) were more likely to have connectivity in contact, grooming, play, proximity, as well as higher quality for proximity. We did not find any evidence for dissimilarity (i.e. heterophily). Stated simply, we found almost uniform homophily for age, kin, rank and sex across all five behaviours, with the following exceptions: age assortativity was absent from grooming and proximity connectivity; rank and sex assortativity were absent from aggression connectivity and contact quality.

##### 3.2.2.4. Other demographic qualities

Although we did not have formal predictions for removals and births, demographic removals during a partition would be anticipated to reduce estimates, as opportunities for socializing are truncated; an effect we all but uniformly found (figure 3). Removal decreased connectivity in aggression, contact, grooming, play and proximity, as well as proximity quality but not contact quality. Birth in the partition before the first available lagged variable was associated with lower connectivity in aggression, grooming, play and proximity, but not contact connectivity; as well as higher quality for contact, yet lower quality for proximity (figure 3).

## 4. Discussion

We found broad predictive power of proximity for the majority of our connectivity and quality across the other behaviours. Contact, too, had predictive power for all the behaviours, except aggression. Our ego traits of age had numerous behavioural associations, which is unsurprising given the largescale behaviour shifts in these behaviours and their expression. Sex and, even more so, rank had complex associations lacking uniformity across behaviours. Generally, assortativity was present for connectivity and quality across the behaviours—defined by sex, rank and age homophily between the ego and their alters. Homophily via kinship was a strong effect in our peer–peer relationships.

### 4.1. Coupled behavioural dynamics

We had the broad expectation that proximity would increase the likelihood of connectivity in all subsequent behaviours. Additionally, that social contact would precede connectivity in play, and that play would meaningfully precede grooming as well as aggression. We found support for all but one of these expectations, with the exception that play connectivity did not precede other behaviours. The connections and quality of proximity in previous time partitions was ubiquitously associated with connectivity and quality in the subsequent partitions of all behaviours, likewise for contact, with the exception of aggression. This dynamic exemplifies the broadscale contribution of proximity to sociality writ-large, while contact contributed to only prosocial behaviours. Play in previous time partitions, however, was not associated with subsequent aggression or grooming connectivity, despite its association with proximity, contact and play itself. In typifying immature male rhesus macaque peer relationships, Colvin [39] determined that the most diffuse (i.e. weakest) relationships were defined by play. Though, the nuance here must be greater, as the number of unique partners was most abundant in our proximity measures.

Our results provide insight as to whether behaviours exhibit coupling within partitions at the level of our animal subjects (egos) and their partners (alters). Within the egos and alters, individual differences in contact and proximity connectivity covaried with each other and with play connectivity, as well as grooming among the egos—indicative of prosocial patterns that bridge behaviours. Neither egos nor alters exhibited consistent patterns of social connectivity among aggression, grooming and play—though we found a weak correlation between egos’ grooming and aggression. We attribute these distinctions due to the complex dynamics of directed behaviours, which are not as salient in undirected behaviours. For example, though ego aggression did not covary with proximity, alter aggression connectivity was negatively associated with proximity connectivity. This incongruence is suggestive of nuanced dynamics whereby egos are incapable of mediating proximity, while the alter themselves or third-parties (i.e. mothers) are mediating access to aggressive alters. That is, peer aggression—at this age—could be beyond egos’ capacity to regulate.

### 4.2. Ego traits

We expected that the ego subjects’ maternal rank would be associated with greater connectivity in all the prosocial behaviours, with uncertain expectations as to aggression connectivity. Egos with high maternal rank, however, had low aggression connectivity, and lower quality for both contact and proximity. Aggression connectivity is more indicative of aggression received (figure 2d). Thus, the low aggression connectivity could be attributable to buffering effects from mothers or close kin, a known phenomenon [11,40–44], though these effects are expected to be reduced as individuals age past 12 months—assuming the birth of a younger sibling [11]. Importantly, even high maternal ranked infants are unlikely to initiate challenges outside of their age-sex class [23]; which our data supported via age—but not sex—homophily for aggression. The lower quality estimates could be attributable to either simple differences in degree or due to a necessitated or intentional social focus among lower ranked individuals. For the former explanation, individuals with higher degree had greater penalization for their versatility indices through normalization, relative to individuals with lower degree. For the latter explanation, lower ranked individuals may be more focused in their socialization (i.e. investing heavily into particular individuals) due to a structural lack of connectivity or through a mechanistic selection process heretofore underexplored. We cannot resolve between these two alternative explanations; raw contact degree was correlated with rank (*r* = 0.29, 0.30 and 0.48 for partitions 2, 3 and 4, respectively) while raw proximity degree varied in its association with rank (*r* = −0.23, 0.08 and 0.50 for partitions 2, 3 and 4, respectively).

We expected sex differences, with male subjects having higher play connectivity, but lower grooming connectivity. Only one of these predictions was supported: males had higher play connectivity, and also proximity connectivity. In hindsight, this dynamic was unsurprising given the associations between egos’ proximity and play random effect estimates, as well as lagged proximity with play, even if unexpected. Grooming connectivity was not lower among males, but this might be attributable to our focus on the full study period, rather than pre-pubescence when grooming sex differences are expected to be more pronounced [7].

Finally, we expected that ageing would correspond with increased aggression and grooming connectivity, but decreased contact, play and proximity connectivity. Our model’s outcomes supported these expectations. Our GAMMS of rates and counts, however, reveal nuanced developmental changes. Contingent on the measure, numerous behaviours had nonlinear relationships with month of development. These changes probably coincided with both developmental and demographic milestones. For example, the count of proximity partners exhibited two ‘bumps’ at approximately 12 and 24 months that likely coincided with birth seasons (figure 2).

### 4.3. Assortativity

We expected and found homophily for kinship across all behaviours. This was true for any matrilineal kinship, but was more pronounced if an ego and their alter were siblings. Such homophily can be contextualized as kin bias [45]. We note, however, that our study group’s structure, whether due to their habits or captive circumstances, was relatively stable—i.e. not having a social overthrow. This is relevant as infants have been shown to exhibit plasticity in kin bias according to group size and composition [46].

We expected rank homophily across all behaviours. We found support for this expectation: similarly ranked egos and alters were more likely to have connectivity in contact, grooming, play and proximity; as well as higher proximity quality. As with maternal rank, it is possible that mothers are limiting access to social partners. These effects are expected to be more pronounced in the first 12 months, after which mothers focus on new infants [11]. If this were the sole determining factor, however, we might expect rank homophily with aggression connectivity, which we did not find. Even without new siblings, developing monkeys are likely to be gaining greater social autonomy in social partner selection by 30 weeks [4]. Among rhesus macaques, rank distance is a defining characteristic that provides independent contributions to social organization, relative to kin bias [47]. Thus, this effect is biologically expected as independent to the effects of kin homophily and maternal effects of rank—though the interplay of these dynamics and their developmental ‘realization’ is probably a compelling direction for future research.

We expected, and found, sex homophily for play. We had less clear expectations for aggression. We expected homophily for females within aggression, while prior evidence suggested that males shifted in assortativity as they matured [14]. Though we would not readily expect pronounced rank–sex interactions at this age [14]. In support of this, aggression connectivity did not exhibit assortativity with rank and sex. Even so, we did not have interaction effects in our model, and so we could not interrogate whether assortativity in connectivity shifted by age. If males and females exhibited distinct patterns of assortativity or if sex assortativity is shifting into maturity, then our models would not be flexible to these changes. We did, however, find an overall effect of sex homophily for connectivity in contact, grooming, play, proximity, as well as higher quality for proximity. Thus, aggression and contact quality violate the general rule of sex homophily during pre-pubescence. Interestingly, sex and rank had identical patterns of assortativity across behaviours.

We found age homophily across all but two of our measures: connectivity for aggression, contact and play; as well as higher contact and proximity quality; nearly in line with our expectations about ubiquitous age homophily. The lack of proximity homophily was unexpected, given our observed associations between play and proximity. We note, also, that age differences are lopsided during earlier partitions, as alters can only be the same age or older than our ego subjects. Thus, heterophily with younger individuals could be present in the later partitions yet not influence the total study period, as observed elsewhere [8,21].

### 4.4. Comparing connectivity and quality

The nature of our data precluded comparisons between connectivity (whether a bond existed between an ego and their alter) and quality (the relative investment in that bond) across all behaviours. We attributed this to sparse networks for many of our directed behaviours relative to our undirected measures. Even so, we can compare trends between connectivity and quality for contact and proximity.

For contact, even after passing the threshold of connectivity, individuals still had variation in their investment in conspecifics according to age and kinship. This was not true, however, for age and sex. This is an interesting point for future research because, for proximity quality, these two variables were still relevant. Also of interest was that maternal rank, for both contact and proximity, was more important for quality rather than connectivity. While it is tempting to attribute this to the size (degree) of ego networks influencing normalization of versatility, connectivity should account for these differences. Our study focused on broad ego and alter traits, yet more nuanced or latent dimensions that influence sociality and have their own developmental trajectories (i.e. personality) [29,32,48], could help clarify unexplained variation in relationship connectivity and quality.

### 4.5. Limitations

Our model performed well across measures of validity and adhered to sociobiological expectations, while providing insight into heretofore underexplored variables. Even so, we note some challenges towards elucidating the dynamics of this system. We aimed to model a complex system with temporally and functionally coupled dynamics (i.e. relationship formation across type and social behaviours). Due to this focus on process, our ability to derive true causal inference is not of foremost focus. This limitation is because we opted to model complexity of the system, at the cost of simplifying to one or two major contributing variables (e.g. whether kinship is of greater importance that social dominance rank). Naturally, there is also great utility in the latter approach, while also accommodating for the complex interconnected components of this system.

## 5. Conclusion

We principally focused on connectivity using an approach that prioritized comparability between diverse behaviours that show developmental variation in their initiation and reception. With evidence from our behavioural dynamics, it was clear that proximity is indicative of general sociality as one might expect, yet contact could be more indicative of prosociality (figures 3 and 4). This is important because contact sitting can be used at early age where more primate-typical measures of prosociality (i.e. grooming) are produced at a very low rate (figure 2). As with prior work, homophily—or similarity principles—were strongly predictive of most behavioural dynamics [49]. In humans, children are able infer relationship status based on such principles [50]. Social dynamics thus emerge early and with complexity that, even in nonhumans, exhibits variability across contexts. Social relationships are multifaceted, and there are nuanced distinctions between connecting with social partners and investing in them.

## Data Availability

The necessary data and analyses to replicate the results presented in this manuscript are available online [51]. Supplementary material is available online [52].
